# Evolution of agricultural biotechnology is the paradigm shift in crop resilience and development: a review

**DOI:** 10.3389/fpls.2025.1585826

**Published:** 2025-06-19

**Authors:** Muhammad Riaz, Erum Yasmeen, Bilal Saleem, Muhammad Khalid Hameed, Maryam Thani Saeed Almheiri, Reem Omar Saeed Al Mir, Ghalia Alameri, Jwaher Salem Khamis Alghafri, Mayank Anand Gururani

**Affiliations:** ^1^ Department of Plant Sciences, School of Agriculture and Biology, Shanghai Jiao Tong University, Shanghai, China; ^2^ National Institute of Genomics and Advance Biotechnology, National Agriculture Research Centre, Islamabad, Pakistan; ^3^ Biology Department, College of Science, United Arab Emirates University, Al Ain, United Arab Emirates; ^4^ Chemistry Department, College of Science, United Arab Emirates University, Al Ain, United Arab Emirates

**Keywords:** molecular breeding, food security, genome editing, climate resilience, sustainable agriculture, artificial intelligence

## Abstract

The dual challenges of climate change and population growth have intensified both biotic and abiotic stresses on crops resulting in disruptions of water dissipation patterns, lessen growth, yield, productivity and food security. Therefore, smart and sustainable agriculture practices for climate resilient and high yielding crops is the need of time. For this purpose, Innovation in biotechnological strategies is essential for sustainable agricultural development. Traditional breeding techniques have evolved through molecular approaches like marker-assisted selection (MAS) and quantitative trait loci (QTL) mapping, which accelerate the identification of trait-specific improvements. Mutational breeding, although effective in generating genetic diversity but lacks the precision, accuracy and effectiveness. Transgenic breeding allows for the transfer of beneficial genes across species, but recent advancements have shifted focus toward more refined approaches, such as RNA interference (RNAi) and genome editing tools like CRISPR-Cas9. These technologies enable precise, controlled genetic modifications to enhance traits like stress tolerance, disease resistance, and nutritional content. The integration of cutting-edge multi-omics platforms, including transcriptomics, proteomics, metabolomics combined with robust artificial intelligence (AI) based methods has revolutionizing crop genome elucidation. AI-driven analysis of large-scale biological data has revealed intricate genetic networks and regulatory pathways that underpin stress responses, growth, yield and genetics circuit patterns. These innovations in biotechnology from conventional breeding to advanced data-trait elucidation integrated methods are pushing the boundaries of climate resilient and next generation crop development. This review focused on the future of resilient and sustainable agriculture that lies in the convergence of conventional and molecular breeding, biotechnology approaches and AI’s driven strategies that enabling scientists to understand the genomics circuits of crops. These next generationally evolved crops bridging gaps from laboratory to field application with reduced reliance on chemical fertilizers, lessen yield gaps, climate resilience and promising nutritional enrichment. Such crops thrive under harsh environment paving the way for resilient and sustainable crop system development in constantly populating and warming ecosystem.

## 1 Introduction

**Figure 1:**
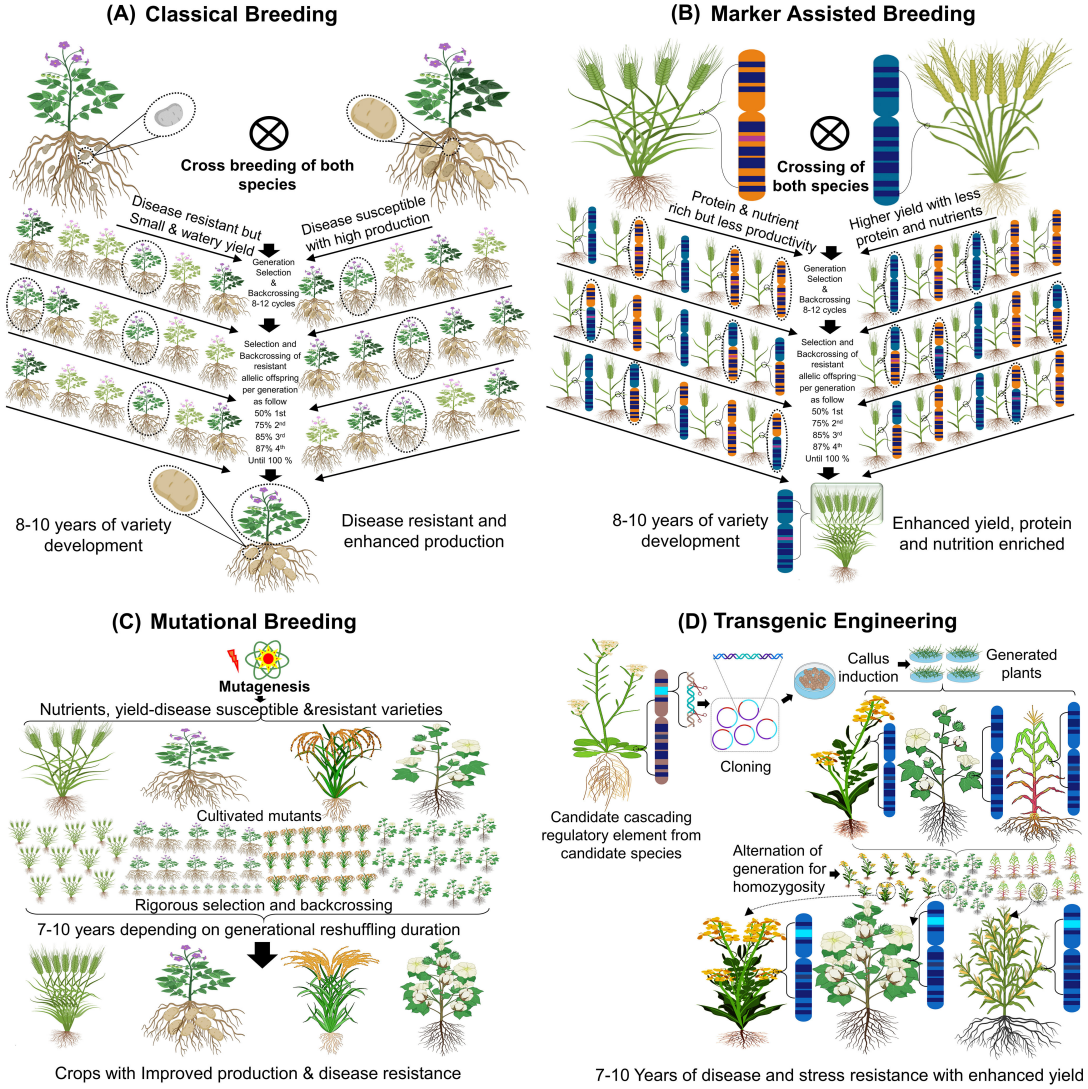
Stress and disease resilient high yielding crop development. **(A)** Selecting and cross-breeding of potato to improve fruit and disease traits through natural variation. **(B)** Molecular marker breeding to select plants with yield, nutrient and protein rich genetic traits. **(C)** Inducing genetic mutations by radioactive material for yield, nutrient and disease resistant trait. **(D)** Inserting foreign genes or manipulation of genes within the genome to develop nutrient rich, disease tolerant, high yielding and resilience crops.

Agriculture is at a crossroads, confronting unprecedented challenges posed by a rapidly growing global population and increasingly erratic climate patterns. Biotic and abiotic stressors including extreme temperature, drought, soil salinity and flooding now severely devastate crop yield, quality, and food security. These environmental disruptions compromise ecosystem stability and jeopardize life on Earth (Davis et al., 2021; Lesk et al., 2022). Addressing these multifaceted challenges necessitate transformative approach of advanced biotechnologies by integrating traditional practices with cutting-edge innovations in crop improvement and their real-world application can be proven by significantly field validation (**Figure 1**). While traditional breeding has historically underpinned agricultural advancement and are now getting apparent in era of climatic instability and escalating global food security. Climatic Implications and limitation are insisting the researchers for developing advanced genetics tool aimed at enhancing crop resilience, productivity, nutritional value and sustainable agriculture (Yu and Li, 2022). Classical breeding including marker-assisted selection (MAS) and quantitative trait loci (QTL) mapping enabling the precise identification and propagation of desirable traits (**Figure 1**). There are several crops with agronomic superiority for tolerance to different stress conditions based on QTLs. Surprisingly, Identified QTLs were failed in actual field research of versatile climatic pressure including barley for yield related characteristics (Genievskaya et al., 2025). This indicates the importance of counter check for laboratory-based breakthroughs and complementation with field trials. Emergence of mutagenesis breeding opened new possibilities to generate genetic diversity for improving stress tolerance in crops. The unexpected phenomenon of wheat yield reductions was observed from 10-28% while performing height and drought related function accordingly after treatment to gamma radiations. Mutagenesis accomplishments for trait characterization required repeatedly seasonal diversity, multiple geological location testing and critical legislatory framework along with molecular correlation (Ahumada-Flores et al., 2021). Transgenic approaches, particularly RNA interference (RNAi), have been instrumental in silencing deleterious genetic circuits but have shown compromised response in variable field trials. Survey of 5 years for RNAi edited rice grown in Asian temperate environment shown effective yield but failed in tropical region with 30-40% efficiency (Davidson and Mccray, 2011; Tardin-Coelho et al., 2025). Genome-editing technologies including zinc finger nucleases (ZFNs), transcription activator-like effector nucleases (TALENs) and CRISPR-Cas9 systems have redefined the scope of crop genetic modification. Experimental to commercial application of CRISPR derived crops revealed challenges and promises including drought tolerance and sensitivity issue in various conditions conclusively highlighting epigenetic role (Wang et al., 2017). There are success stories for CRISPRCas9 and its variant based editing in Rice (*DST, SPL10, NAC041* for salt tolerance and *LCT1, HAK1, PRX2, NRAMP5, ARM1* for abiotic stress tolerance) (Rahman et al., 2022; Kumar et al., 2023), Maize (*ARGOS8* for drought tolerance and *PAP1* for flavone content improvement) (Rasheed et al., 2023; Mackon et al., 2023), Wheat (*DEP1* and *LOX2* for Nitrogen use efficiency) (Li et al., 2022a; Bharat et al., 2020) and tomatoes (*AGL* and *CBF1* temperature and drought tolerance) (Wang et al., 2019; Zhao et al., 2021). CRISPR’s advancements i.e., base and prime editing paving way to unparalleled precision in correcting genetic sequences (Ahmar et al., 2024; Pacesa et al., 2024). Prime editing, a groundbreaking innovation and establishing a new frontiers in crop genetic engineering by targeted modifications without inducing double-stranded DNA breaks (Li et al., 2024). These tools have significantly accelerated the development of stress-resilient crops with enhanced yield potential and entered to legislation period in EU and US. The convergence of multi-omics technologies, synthetic biology and artificial intelligence (AI) heralds a new era in crop science. Integrating genomic, transcriptomic, proteomic and metabolomic enabling researchers to elucidate and enhance stress resilience at the molecular to field level (Lee et al., 2012). Biosynthetic engineering enabling reconstruct and optimize novel biosynthetic pathways to with improved traits, boosting both yield and nutritional content. Nitrogen fixation and use efficiency of cereal crops including maize can be enhance by genetic circuit optimization with soil microbiome (Wen et al., 2021). AI-driven predictive models are further refining these efforts and offering unprecedented insights into plant-environment interactions (Gao, 2021; Sun et al., 2022). Landmark investigation coupling artificial intelligence and machine learning models with phenomics data from various crops including rice, wheat and maize predicted accurate yield in variable climatic condition over the globe (Xu et al., 2022). In this review, we try to address the remarkable evolution of agriculture biotechnology with emphasis on conventional crop improvement to modern laboratory generated molecular analysis, integration of artificial intelligent multi-omics platforms and essential role of field validation in transforming data driven breakthroughs into sustainable development and solutions. The review predominantly highlights critical barriers for laboratory to farmer and field research results validation to better understand biotechnological potential of crops. The current era of potential development required to boost agriculture and resilient crop biology by artificial intelligence based cutting edge technology integration. Such integration would bring robust laboratory trials, field testing and creating continuous feedback mechanism between stakeholder and policy makers to implement agriculture governance. Rarely such advanced approaches are hope to make us withstand against extreme climate and develop true resilient crops to meet nutritional demand and food security of a growing 21^st^ century.

## 2 Environmental cues limiting crop productivity

The ecosystem governed by competitive selection pressures, the challenges of survival, adaptation, and resource availability are naturally amplified. In the current era of overpopulation and erratic climate fluctuations, stress influencers such as extreme temperatures, drought, barren soils, and floods have drastically reduced both the yield and quality of vital food supplies with denatured soil structure. While traditional crop breeding methods have been instrumental in agricultural development, they are time-intensive and often insufficient to address the escalating demands for productivity, disease resistance, and biomass generation (Zhang et al., 2018). This highlights the pressing need to harness modern and safe biotechnological tools for developing stress-resistant and high-yielding crops. Despite significant advancements, achieving robust resilience to biotic and abiotic stresses remains a formidable challenge. The molecular and physiological responses of crops to stressors are intricate and multifaceted, often involving overlapping and interdependent pathways (Zhang et al., 2017, 2020). The simultaneous activation of different stress-response pathways can exacerbate declines in yield, quality, and crop texture, further complicating breeding efforts. Decoding these complex stress tolerance mechanisms requires a collaborative effort from plant breeders, genetic engineers, and molecular researchers. Emerging technologies, such as artificial intelligence (AI), machine learning, deep learning, and systems biology, are revolutionizing the analysis of vast datasets to uncover the genetic and molecular pathways underpinning stress resilience (Seleiman et al., 2021). These tools enable researchers to dissect plant physiological, molecular, and metabolic responses to stress, providing critical insights for sustaining and enhancing resilience traits in dynamic environments. Recent progress in molecular and evolutionary biology has yielded cultivars with enhanced tolerance to diverse biotic and abiotic stressors (Zhang et al., 2023). Innovative genome-editing technologies, such as CRISPR-Cas9 systems, have emerged as powerful tools for creating crops with superior resilience and improved productivity. These advancements have paved the way for engineering plants capable of withstanding multiple stressors, ensuring stable food production amid growing global challenges. Understanding the intricate molecular mechanisms of stress tolerance and leveraging biotechnological innovations hold immense potential for the future of agriculture. By integrating cutting-edge genomic tools with insights from systems biology, it is possible to cultivate resilient, high-quality crops that thrive under adverse environmental conditions (Lohani et al., 2022). Such innovations are crucial for safeguarding food security and meeting the nutritional demands of a rapidly expanding population.

## 3 Plant genetics and breeding approaches for productivity and environmental resilience

Classical breeding, also referred to as conventional or traditional breeding, involves the development of new cultivars by introducing desired traits, such as stress tolerance and high yield, through crossbreeding or hybridization (**Figure 1**) (Wuest et al., 2021). The domestication of plants via conventional breeding began in the early 20th century, spearheaded by Gregor Mendel, the father of classical genetics, who developed the first high-yielding and nutritious crop cultivars (Van Dijk et al., 2018). In the 1960s, N. Borlaug advanced this field by producing high-yielding wheat and rice varieties (Swaminathan, 2009). Classical breeding focuses on selecting specific traits or phenotypes, such as biotic and abiotic stress resilience or high yield, and linking these traits to genetic changes resulting from extensive crossing between closely related species over successive generations. Stabilizing these traits often requires 5–6 cycles of selfing, with the process of developing stable, high-performing cultivars taking 12–15 years (Gao, 2021; Husaini, 2022). While classical breeding has successfully produced disease-resistant and high-yielding crops such as potatoes (*Sli* gene elucidation (Eggers et al., 2021)), salt-tolerant wheat, and drought-tolerant barley (As explained in **Figure 1**). This limitation has prompted the development of advanced technologies to accelerate crop improvement.

### 3.1 Marker-assisted selection and quantitative trait loci for crop improvement

Marker-assisted selection (MAS) utilizes molecular markers linked to specific traits, such as disease resistance or stress tolerance, to enhance breeding efficiency. MAS integrates classical genetics with molecular biology, relying on phenotypic, biochemical, or DNA markers to select for specific traits (Su et al., 2019; Xu et al., 2022). It has been instrumental in improving complex traits such as stress tolerance and disease resistance, especially with the advent of high-throughput genotyping and association mapping. Quantitative trait loci (QTL) are genomic regions associated with phenotypic traits. High-throughput phenotyping technologies link genetic information to specific traits, aiding in the identification of QTLs governing stress tolerance, yield, or disease resistance (Tardieu and Tuberosa, 2010). Techniques such as linkage disequilibrium (LD) mapping and genome-wide association studies (GWAS) enable precise identification of single nucleotide polymorphisms (SNPs) associated with traits like heat stress tolerance (Younessi-Hamzekhanlu and Gailing, 2022). Combining MAS, QTL, and GWAS accelerates the development of resilient crop varieties (Scott et al., 2020; Luo et al., 2022).

### 3.2 Mutational breeding for better traits

Mutational breeding introduces genetic variations through chemical, physical, or biological mutagenesis. Techniques such as gamma or X-ray irradiation, chemical mutagens like ethyl methane sulfonate (EMS), and site-directed mutagenesis by Agrobacterium T-DNA transformation have been used to modify traits related to yield, disease resistance, and stress tolerance (Jankowicz-Cieslak et al., 2017). One prominent reverse genetics approach, Targeted Induced Local Lesions in Genomes (TILLING), identifies mutations in specific genes. Coupling TILLING with next-generation sequencing (NGS) has facilitated the discovery of allelic variations crucial for stress resilience (Toppino et al., 2022). Mutational breeding (**Figure 1**), has successfully produced crops like rice, tomato, cotton, and wheat with improved traits (Liu and Zhang, 2022).

### 3.3 Transgenic manipulation and engineering for quality traits

Transgenic engineering integrates molecular biology techniques to create crops with desired traits by introducing specific genes or genomic elements into the target plant. Advances in recombinant DNA technology, such as GATEWAY cloning, Gibson assembly, and seamless ligation-independent cloning, have enabled the precise manipulation of plant genomes (Furmanek-Blaszk et al., 2009). Agrobacterium-mediated transformation and biolistic methods are the most widely used techniques for crop genetic engineering. Agrobacterium tumefaciens delivers T-DNA constructs into the host genome, allowing for the stable expression of transgenes for traits like stress tolerance and higher yield (Hoekema et al., 1983). Biolistic transformation, or particle bombardment, directly introduces DNA into plant cells, bypassing genotypic barriers, and has been used to develop crops like wheat, maize, and rice with enhanced traits (Garvin et al., 2008; Zhi et al., 2022). Both methods have contributed to developing transgenic crops capable of thriving under adverse environmental conditions, ensuring higher productivity and resilience. The flexibility of these techniques allows the use of diverse regulatory elements, such as promoters and enhancers, to achieve trait-specific expression, further advancing crop improvement efforts.

## 4 RNA interference for controlling negative traits and crop improvement

**Figure 2:**
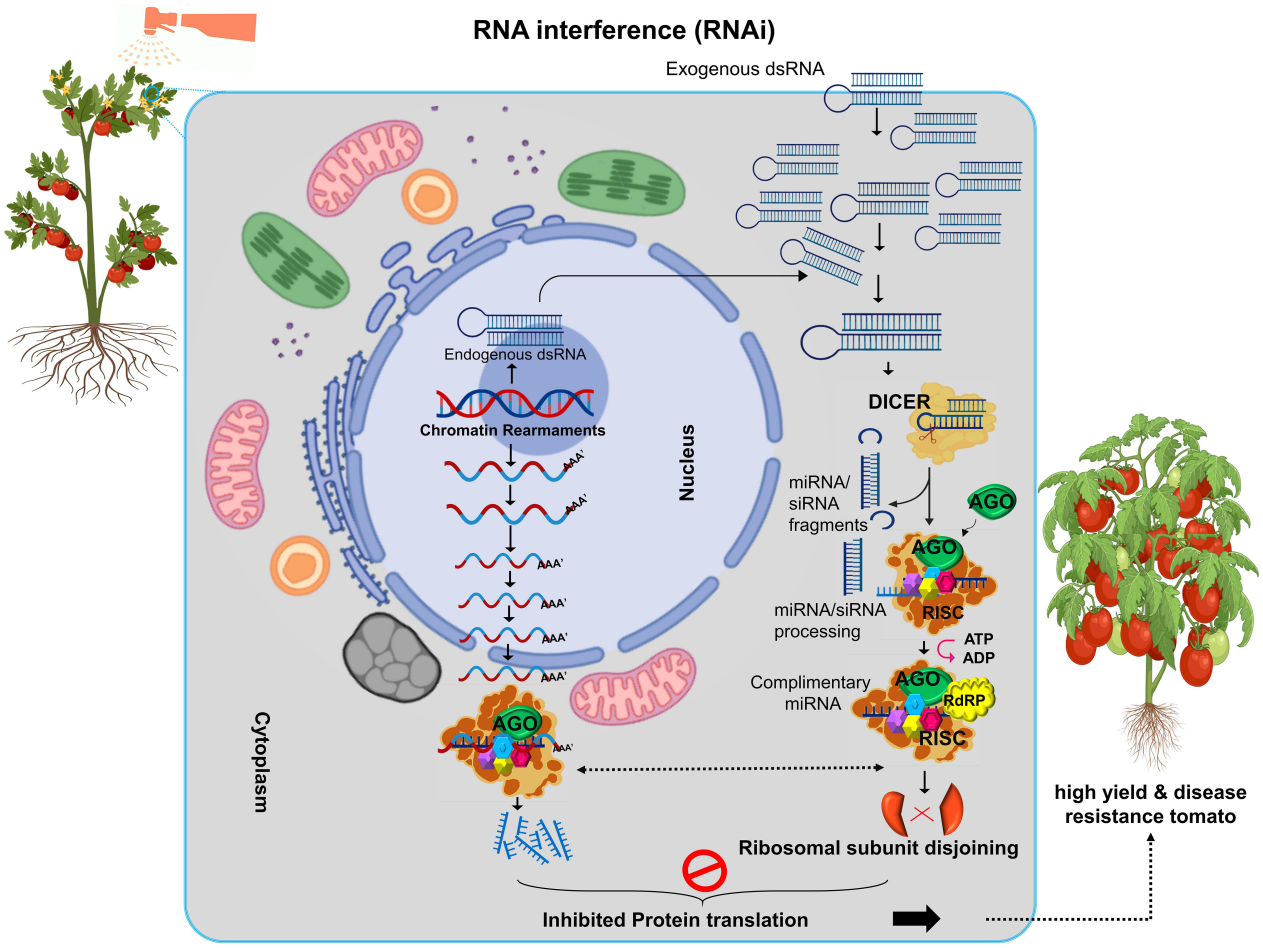
RNA interference (RNAi) technology used in crops to silence specific genes, enhancing resilience to both internal (endogenous) and external (exogenous) stresses. RNAi helps plants withstand environmental challenges like drought, heat and pests, making it a valuable tool for breeding stress-tolerant crops.

RNA interference (RNAi) is a breakthrough mechanism for downregulating, silencing, upregulating, or controlling gene expression in a specific manner (**Figure 2**). This phenomenon was first identified by R. Jorgensen in 1990 while attempting to enhance the color of petunia flowers by introducing multiple copies of the chalcone synthase (Chls A) gene. Instead of producing dark purple flowers, the experiment yielded white and patchy flowers, a result of gene silencing at homologous, endogenous, and exogenous loci (Napoli et al., 1990). The pivotal discovery of RNAi as a molecular mechanism was made by Fire and Mello in 1998, who observed that double-stranded RNA (dsRNA) silenced homologous genes in C. elegans (Fire et al., 1998; Tabara et al., 1999). Their work earned them the Nobel Prize in Physiology or Medicine in 2006. They demonstrated that dsRNA triggered gene silencing via a sequence-specific mechanism, leading to translational inhibition of target mRNA (Devanapally et al., 2021). RNAi operates through sequence complementarity between dsRNA and the target mRNA. This specificity inhibits gene expression by cleaving mRNA into fragments, serving as templates for RNAi activity (Gu et al., 2012). The molecular mechanism comprised of Dicer-like Proteins (DCL), in which RNAse III enzymes process dsRNA into small interfering RNAs (siRNAs), typically 18–24 base pairs in length. RNA-Induced Silencing Complex (RISC) in which siRNAs guide this protein complex, containing Argonaute (AGO) proteins, to bind complementary mRNA sequences. The activated RISC-miRNA complex cleaves or represses the target mRNA, halting translation. Then, RNA-Dependent RNA Polymerase (RdRP) enzyme amplifies the silencing signal, ensuring sustained gene suppression. RNAi results in either post-transcriptional gene silencing (mRNA degradation) or transcriptional repression via chromatin rearrangements (Sontheimer, 2005; Wei et al., 2023). RNAi has revolutionized genetic engineering for developing stress-tolerant, high-yield, and disease-resistant crops. RNAi enables precise regulation of gene expression by introducing dsRNA complementary to specific target genes. The wide accepted benefits of RNAi for crop improvement includes, the development of heat, drought, salt resistant crops and Improved resistance to pathogens and pests. Metabolic pathway redirection by enhancing production of desired metabolites and improving crop quality. There are several methods for RNAi induction in Plants mainly include virus induced gene silencing (VIGS) that utilize viral vectors to introduce dsRNA for gene silencing. Agrobacterium-Mediated Transformation that deliver RNAi constructs into plant genomes via Agrobacterium tumefaciens. Direct Spray/Biolistic Bombardment involved direct application of RNAi molecules or constructs to plant tissues. These methods have enabled significant advancements in understanding gene function and enhancing crop resilience to environmental challenges (Chung et al., 2021; Lopez-Gomollon and Baulcombe, 2022).

## 5 Genome editing revolutionizing crop resilience via (ZFNs), (TALENs) and (CRISPR)

**Figure 3:**
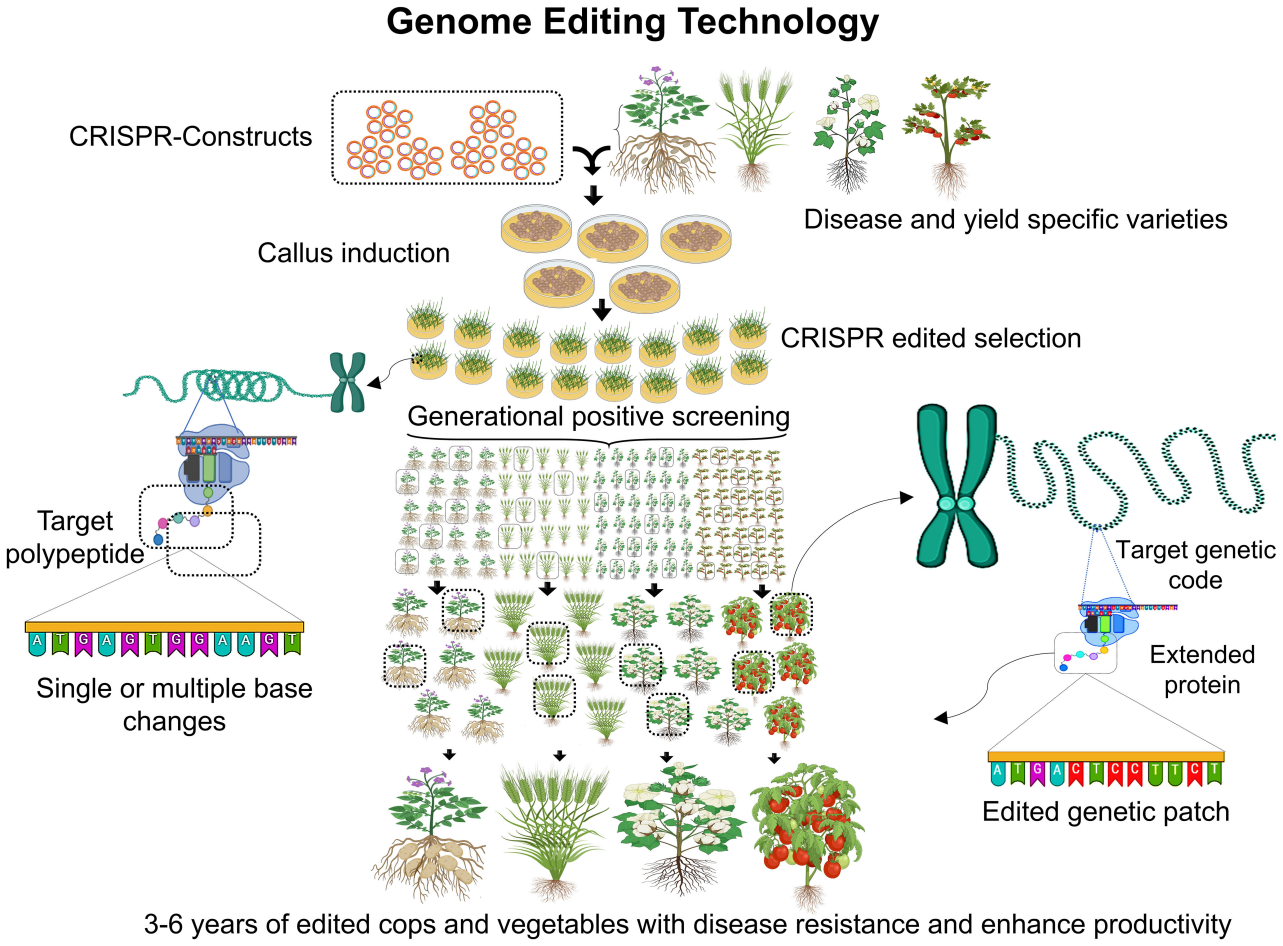
An illustration of CRISPR-based genome editing for resilient and targeted crop traits. The process begins with the creation of CRISPR constructs, followed by callus induction and the selection of CRISPR-edited plants. Generational positive screening ensures disease-resistant and high-yield crop varieties through precise genetic modifications, including single or multiple base changes and targeted genetic patching. The timeline for producing enhanced crops and vegetables with improved productivity and resistance ranges from 3 to 6 years.

Genome editing technologies (**Figure 3**), have gained wide spread acceptance for enhancing crop traits, disease resistance, food production, and environmental adaptability. These techniques, which date back to the 1980s, have evolved significantly over the years, with advancements continually improving precision and efficiency in molecular biology and crop development. Various genome manipulation techniques enabled gene insertion, deletion and overexpression to enhance crop traits for resilience and superior performance. There are several examples of CRISPR-Cas9 and its advanced variant-based editing of traits in rice, maize, wheat and other domestic to model crops (**Table 1**). These genetically modified organisms (GMOs) can exhibit improved traits but also raise concerns, such as potential allergenicity or microbial resistance due to genomic reshuffling (Rozas et al., 2022). The prominent genome editing technologies are zinc finger nucleases (ZFNs), transcription activator-like effector nucleases (TALENs) and clustered regularly interspaced short palindromic repeat (CRISPR) with various examples of varieties and gene trait testing are documented (**Table 1**). CRISPR stands out for its ability to induce desired traits without introducing foreign genetic material, making it more widely accepted. However, each technique has its advantages and limitations, contributing uniquely to developing customized crops with higher yields, stability, and tolerance to environmental and physiological stresses (Lee et al., 2016; Ricroch, 2019). ZFNs were introduced in the 1990s by Sangamo Biosciences which hold intellectual property rights (Scott, 2005). This technique relies on restriction enzymes composed of zinc finger DNA-binding domains and nonspecific cleavage motifs from FokI endonuclease. A single zinc finger unit recognizes 4–6 base pairs, with a pair recognizing up to 24 base pairs, creating double-stranded breaks (DSBs) through FokI dimerization. Crops like *Arabidopsis thaliana* and *Zea mays* have been edited using ZFNs, resulting in herbicide tolerance, enhanced yield, and resistance to biotic and abiotic stresses (Ahmar et al., 2020). However, ZFNs face limitations, including high costs, time-consuming development, and off-target mutations, reducing their accuracy and efficiency. These shortcomings prompted the development of newer genome editing technologies (Urnov et al., 2010). TALENs, discovered by D.F. Voytas, offer more accurate genome editing than ZFNs. These enzymes use transcription activator-like effector (TALE) proteins fused with FokI nucleases for DNA cleavage. Unlike ZFNs, TALENs can edit longer DNA sequences with greater specificity and reduced off-target effects (Li et al., 2022b). TALE proteins consist of a DNA-binding domain with tandem repeats of 34 amino acids, specifying target recognition. Nuclear localization signals. Activation domains for transcriptional activity. TALENs have been successfully used to edit rice (*Oryza sativa*), achieving biallelic modifications in a single generation. This method is preferred for its cost-effectiveness, adaptability, and ability to target DNA regions without protospacer adjacent motif (PAM) site restrictions (Yee, 2016; Iqbal et al., 2020).

**Table 1:** Trait based improvement of different model and domestic crops by conventional and advanced genome editing methods.

Editor Type	Architecture	Crop Species	Target gene	Improvement Trait	References
RNAi	RNAi construct assembly	Zea mays	*ZmCry3A, Cry Proteins (Bt Toxins)*	Disease resistance	(Gassmann et al., 2014)
ZFNs	Zinc finger and non-specific FokI nuclease domain, dimeric protein	*Zea mays*	*MS26, PAT*	Sterility and herbicide tolerance	(Ran et al., 2018)
	Zinc finger and non-specific FokI nuclease domain, dimeric protein	*Nicotiana tobaccum*	*GUS: NPTII*	Chromosomal breakage identification	(Baltes et al., 2014)
	Zinc finger and non-specific FokI nuclease domain, dimeric protein	*Gossypium hirsutum*	*EPSPS*	Herbicide resistance	(Hussain et al., 2021)
	Zinc finger and non-specific FokI nuclease domain, dimeric protein	*Arabidopsis thaliana*	*CP2*	Insect resistance	(Khan et al., 2018)
TALENs	TALE DNA binding and non-specific FokI nuclease domain, dimeric protein	*Oryza sativa L*	*OsBADH2*	Aromatic rice development	(Hui et al., 2022)
	TALE DNA binding and non-specific FokI nuclease domain, dimeric protein	*Solanum tuberosum*	*VInv/Pain-1 encoded vacuolar invertases, ALS*	Halting sugar reduction mechanism, Herbicide resistance	(Ahmad et al., 2022)
	TALE DNA binding and non-specific FokI nuclease domain, dimeric protein	*Oryza sativa L*	*SWEET14, TMS5*	Disease and heat tolerance	(Tiwari and Lata, 2019)
	TALE DNA binding and non-specific FokI nuclease domain, dimeric protein	*Saccharum officinarum L*	*Caffeic acid-O-methyltransferase (COMT)*	Lignin reduction	(Martarello et al., 2023)
CRISPR-Cas9	crRNA, tracrRNA, Cas9 protein	*Oryza sativa L*	*OsLCT1, OsHAK1, OsPRX2, OsNRAMP5, OsARM1*	Abiotic stress and transporting role	(Rahman et al., 2022)
	crRNA, tracrRNA, Cas9 protein	*Oryza sativa L*	*GBSS*	Amylose enhancement	(Ying et al., 2023)
	crRNA, tracrRNA, Cas9 protein	*Oryza sativa L*	*OsDST, OsSPL10, OsNAC041*	Salt tolerance	(Kumar et al., 2023)
	crRNA, tracrRNA, Cas9 protein	*Oryza sativa L*	*OsTB1*	Thermotolerance	(Kouhen et al., 2023)
	crRNA, tracrRNA, Cas9 protein	*Lycopersicum esculentum*	*SlCBF1*	Chilling tolerance	(Zhao et al., 2021)
	crRNA, tracrRNA, Cas9 protein	*Gossypium hirsutum*	*ALARP*	Fiber elongation	(Zhu et al., 2021)
	crRNA, tracrRNA, Cas9 protein	*Lycopersicum esculentum*	*NPR1, SIMAPK3*	Abiotic stress tolerance	(Ali et al., 2022)
	crRNA, tracrRNA, Cas9 protein	*Lycopersicum esculentum*	*AGL6*	Thermotolerance	(Wang et al., 2019)
	crRNA, tracrRNA, Cas9 protein	*Zea mays*	*ARGOS8*	Drought tolerance	(Rasheed et al., 2023)
	crRNA, tracrRNA, Cas9 protein	*Lycopersicum esculentum*	*SlPL*	Gray mould resistance	(Shi et al., 2023)
	crRNA, tracrRNA, Cas9 protein	*Arabidopsis thaliana*	*OsT2, WRKY4, OXP1*	Drought and salt tolerance	(Sami et al., 2021)
	crRNA, tracrRNA, Cas9 protein	*Vitus vinifera*	*PDS, MLO-7, WRKY52*	Albinism trait, Biotic stress tolerance	(Ren et al., 2021)
	crRNA, tracrRNA, Cas9 protein	*Zea mays*	*PAP1*	Increase flavone content	(Mackon et al., 2023)
	crRNA, tracrRNA, Cas9 protein	*Oryza sativa L, Vitus vinifera*	*OsEPFL9*	Stomatal density (SD) control	(Lu et al., 2019)
	crRNA, tracrRNA, Cas9 protein	*Oryza sativa L*	*Hd2, Hd4, Hd5, EPFL9, ROC5, DEP1*	Stomatal conductance and seed development	(Fang et al., 2021)
BE	APOBEC1‐XTEN‐ nCas9	*Oryza sativa L*	*OsALS, OsDELLA, OsETR1*	Herbicide resistance	(May, 2017)
CBE2/CBE3	APOBEC1‐XTEN‐ nCas9, APOBEC1-XTEN-Cas9(D10A	*Oryza sativa L*	*OsSLR1*	Nitrogen use efficiency	(Roy and Soni, 2021)
CBE	CD-Cas9n-UGI	*Arabidopsis thaliana, Oryza sativa L*	*ALS*	Herbicide tolerance	(Dong et al., 2020)
CBE2/CBE3	APOBEC1‐XTEN‐ nCas9‐UGI	*Oryza sativa L*	*OsSBEIIb, OsPDS*	High amylose	(Yarra and Sahoo, 2021)
CBE2/CBE3, hAID‐CBE3	APOBEC1‐XTEN‐ nCas9/dCas9‐UGI, hAID‐XTEN‐nCas9	*Oryza sativa L, Triticum aestivum*, *Zea mays*	*OsCDC48, OsNRT1.1B, OsSPL14, TaLOX2, ZmCENH3*, *OsFLS2, OsAOS1, OsJAR1, OsJAR2, OsCOI2, OsPi‐D2*	High nitrogen usage efficiency and yield, Herbicide resistance	(Bharat et al., 2020)
CBE2/3/4	PmCDA1‐nScCas9+ +‐UGI‐UGI	*Oryza sativa L*	*OsWaxy, OsEUI1*	Amylose reduction	(Zeng et al., 2020)
CBE3, APOBEC3A	APOBEC1‐XTEN‐ nCas9‐UGI, PTP‐TALE‐L‐nDdA‐ UGI‐PTP‐TALE‐R‐ cDdA‐UGI	*Gossypium hirsutum, Arabidopsis thaliana*	*GhCLA, GhPEBP, 16s rRNA, rpoC1, psbA*	Genetic trait change	(Li et al., 2023)
ABE	TadA-32aa-TadT6.3/7.8/7.9/7.10-32aa-Cas9n	*Arabidopsis thaliana*	*PDS, FT, LFY*	Herbicide tolerance	(Zhou et al., 2021)
ABE	TadAwt + 7–10-nVQRCas9- NLS(ABE-P3)	*Oryza sativa L*	*SPL14,16,17,18*	Yielding character	(Dhakate et al., 2022)
ABE7.10	TadA‐TadA7.10‐ nCas9 (D10A), PmCDA1-nCas9(D10A), APOBEC1-XTEN-dCas9	*Oryza sativa L, Triticum aestivum*,	*OsALS, OsCDC48, OsAAT, OsDEP1, OsACC, OsNRT1.1B, OsEV, OsOD, TaDEP1*	Herbicide resistance, Nitrogen use efficiency	(Li et al., 2022a)
ABE	TadA-32aa-TadT7.10-32aaCas9n	*Arabidopsis thaliana*	*FT, PDS3*	Spliced functional affects studies	(Kang et al., 2018)
ABE	pUC57-APOBEC1-XTEN-n/dCas9-UGI	*Oryza sativa L and Triticum aestivum* *Zea mays*	*OsCDC48*, *OsNRT1.1B, OsSPL14*, *TaLOX2* *ZmCENH3*	Developmental substitution	(Zong et al., 2017)
PE	Sp-PE2, Sp-PE3	*Oryza sativa L*	*OsALS, OsIPA1, OsTB1*	Yield enhancement	(Zhan et al., 2021)
PE	pPE2	*Oryza sativa L*	*OsPDS, OsACC, OsWx, OsALS*	Herbicide tolerance	(Hao et al., 2021)
PE	Sp-PE3, PE4	*Oryza sativa L*	*GFP, APO1, OsACC, OsEPSPS*	Herbicide tolerance	(Tabassum et al., 2021)
PE	pCXPE03	*Lycopersicum esculentum*	*SIGAI, SIALS, PDS1*	Functional efficiency	(Lu et al., 2021)
PE	PE3-DS	*Oryza sativa L*	*OsNR2, OsALS, OsSPL14, OsALS, OsDHDPS*,	Yield enhancement	(Xie et al., 2022)
PE	pH-nCas9-PPE3, PPE2, PP23b	*Triticum aestivum*, *Oryza sativa L*	*TaGW2, TaMLO, TaGASR7, TaDME, TaLOX2, TaUbi10*, *OsAAT, OsALS, OsCDC48, OsDEP1, OsEPSPS, OsGAPDH*	Crop improvement	(Lin et al., 2020)
PE	pPE2max-evoporeQ1	*Oryza sativa L*	*OsCDC48, OsACC*	Herbicide tolerance	(Li et al., 2022c)
PE	EnpPE2	*Oryza sativa L*	*OsALS, OsPDS, OsACC*	Herbicide tolerance	(Lin et al., 2021)
PPE	pH-CBE, pH-nCas9-PPE	*Oryza sativa L*	*OsALS*, *OsCDC48*, *OsCDC48*, *OsGAPDH*, *OsLDMAR*	Editing efficiency	(Jin et al., 2021)
PPE	pH-nCas9-PPE	*Oryza sativa L*	*OsAAT, OsACC, OsALS, OsCDC48), OsDEP1, OsEPSPS, OsIPA1, OsNRT1.1B, OsGAPDH, OsPDS, OsROC5*	Functional substitution efficiency confirmation of PPE	(Lin et al., 2021)
ePPE	ePPE–SpG, pH-ePPE	*Oryza sativa L and Triticum aestivum*	*OsAAT, OsACC, OsALS, OsCDC48, OsDEP1*, *OsEPSP1, OsGAPDH, OsIPA1, OsDMAR, OsNRT1.1B*, *OsODEV, OsPDS, OsROC5, OsPDS, OsALS, OsRDD1, TaDME1, TaGW2, TaLOX2. TaNAC2, TaSBEIIa, TaGRF1, TaGRF4*	Substitutional frequency confirmation	(Zong et al., 2022)

CRISPR/Cas9, the latest and most widely accepted genome editing technique, revolutionized crop improvement due to its simplicity, precision, and affordability (Al-Attar et al., 2011). Unlike earlier methods, CRISPR does not rely on foreign DNA, using the organism’s native machinery to induce or modify gene expression (Ishii and Ishii, 2022). The CRISPR mechanism involves the guide RNA (gRNA), A sequence-specific RNA that directs the Cas9 protein to the target DNA. Cas9 Protein endonuclease introduces DSBs near the PAM sequence (NGG) (Pickar-Oliver and Gersbach, 2019). CRISPR is classified into two main classes in class I involves multiple effector proteins and II, relies on a single effector protein like Cas9, making it more efficient and widely used (Shmakov et al., 2017). Upon introducing DSBs, the cellular machinery repairs the breaks through three primary mechanisms, 1): non-homologous end joining (NHEJ), which is quick repair but prone to indels, causing frameshift mutations (Peterka et al., 2022), 2):Microhomology-mediated end joining (MMEJ) create deletions and chromosomal rearrangements, often independent of Ku proteins or DNA ligase 4 (Stinson et al., 2020). The third is homology directed repair (HDR), which is precise mechanism using homologous templates for error-free repair, primarily active during the S and G2 phases of the cell cycle (Chen et al., 2022). CRISPR has enabled breakthroughs in crop development, allowing researchers to create resistant, high-yield, and stress-tolerant crops. Despite its successes, some sub-mechanisms, like MMEJ, remain under investigation to further enhance its utility (Molla et al., 2021). Genome editing technologies continue to transform agricultural research and crop development. Each method including ZFNs, TALENs, and CRISPR, offers unique contributions to creating resilient and superior crops, addressing global food security challenges in an environmentally sustainable manner.

### 5.1 Base editing to avoid drastic genomic complexity

**Figure 4:**
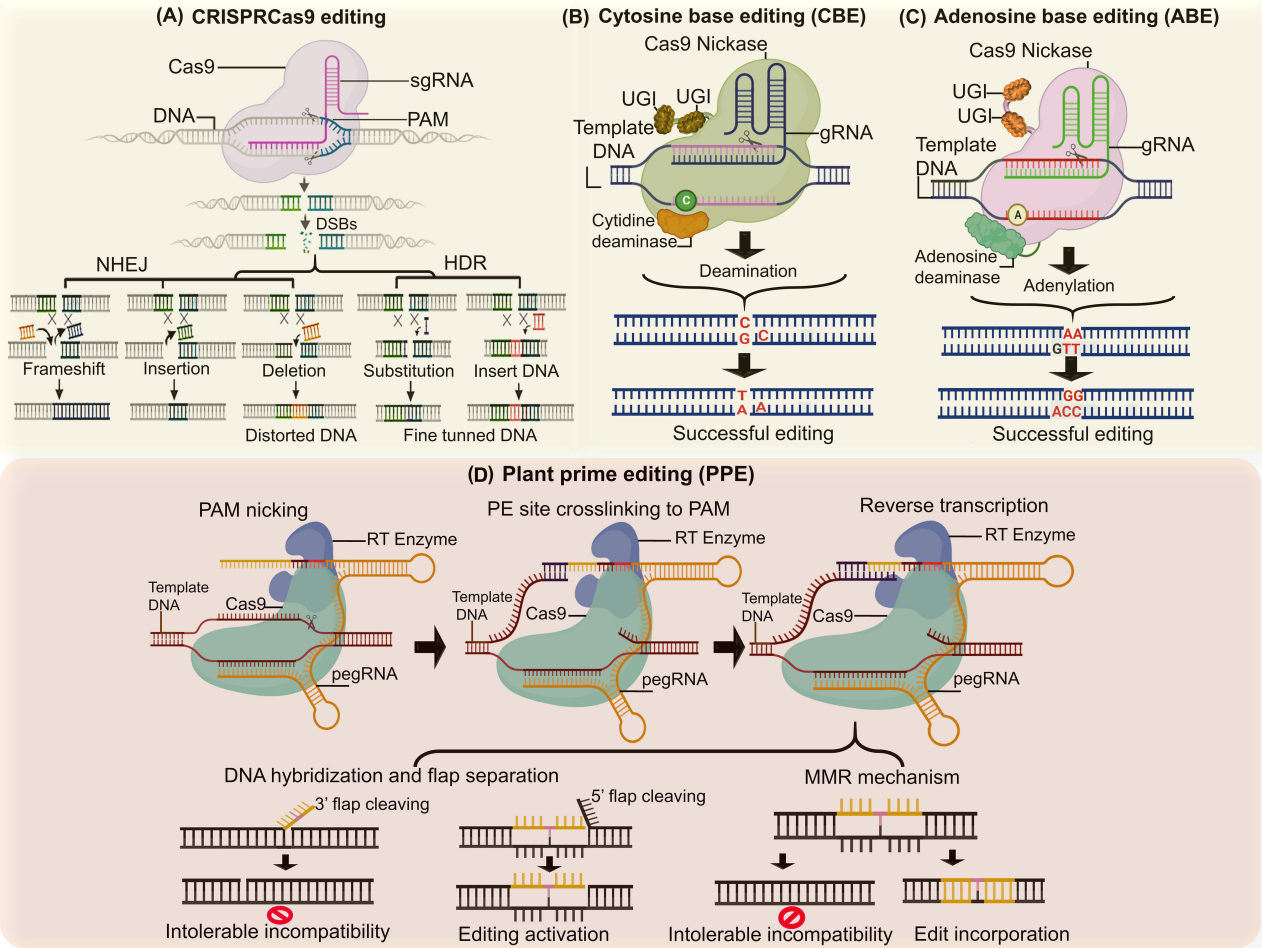
Base editing approaches spanning single base edits to multiple patches or ORF for targeted trait change. **(A)** CRISPR editing enables precise DNA cuts to add, remove or modify genes, facilitating targeted improvements in traits like disease resistance and yield. **(B)** Cytosine base editors convert cytosine (C) to thymine (T) in DNA without cuts, allowing precise alterations of genes linked to development and stress tolerance. **(C)** Adenosine base editors change adenine (A) to guanine (G) without cutting DNA, enabling specific modifications in traits like flowering time and nutrient efficiency. **(D)** Prime editors perform precise edits by inserting, deleting, or altering bases without double-strand breaks and facilitating complex trait enhancements in crops.

The improvement of agronomic traits in crops has been revolutionized by genome editing technologies, particularly through single nucleotide polymorphism (SNP) determination and confirmation (Gao, 2021). Substituting bases offers immense potential for introducing new crop varieties with enhanced traits. Base editing is a transformative molecular tool, enabling precise, programmed modifications to plant genomes (Zhu et al., 2020). Its rapid adoption and global acceptance stem from its precision, accuracy, and functional durability. Base editing achieves permanent and targeted single DNA or RNA base conversions without requiring double-strand breaks (DSBs) or repair mechanisms. This process involves DNA and RNA base editors, which utilize inactive CRISPR-Cas9 modules (dead Cas9, Cas9 nickase, or Cas9 variants) fused with cytosine and adenosine deaminases (Li et al., 2018; Pacesa et al., 2024). In developing new crop traits, base editors are categorized into cytosine base editors (CBEs) (**Figure 4**) and adenosine base editors (ABEs) (**Figure 4**). Cytosine base editors (CBEs) are formed by fusing cytidine deaminase with the inactive domain of CRISPR-Cas9. CBEs induce deamination, converting cytosine (C) to uracil (U), which is recognized as thymine (T) during DNA replication, thereby enabling C.G to T.A substitutions (Neugebauer et al., 2023). Adenosine base editors (ABEs) consist of Cas9 nickase, sgRNA, and transfer RNA (tRNA) adenosine deaminase (TadA), responsible for deaminating adenosine (A) to inosine (I). Inosine is interpreted as guanine (G) during DNA replication, allowing precise A.T to G.C conversions (Rees and Liu, 2018). There are number of examples for editing genes within the crops are reported in by various researchers (**Table 1**).

### 5.2 RNA editing to elucidate developmental perturbation in plants

RNA editing involves post-transcriptional modifications by altering nucleotides in mRNA to produce like plastids and mitochondria. RNA editing in coding sequences of mRNA is evolutionarily stable and functionally significant, often restoring gene functions lost due to mutations (Gommans et al., 2009). This natural repair mechanism, combined with the precision and reduced off-target effects of base editing, makes it a promising tool for developing stress-resilient crops under changing environmental conditions.

### 5.3 Prime editing and editors’ evolution as an additional strategy for smart crops

Prime editing (PE) is an advanced CRISPR-based genome editing tool (**Figure 4**), with higher accuracy and efficiency, coupled with minimal off-target effects that was firstly introduced by David R. Liu’s research group (Chen and Liu, 2023; Huang and Liu, 2023). PE employs a “search and replace” mechanism to modify target sites (Anzalone et al., 2019). It enables four base transitions (e.g., A→G, C→T) and eight transversions (e.g., G→C, A→T) and supports insertions and deletions of 80–40 base pairs (Nelson et al., 2022a). The major components of PE system comprise prime editing guide RNA (pegRNA) that identifies and guides nucleotide replacement. The fusion protein which consists of a Cas9 H840A nickase fused with murine leukemia virus (M-MLV) reverse transcriptase. Single guide RNA (sgRNA) directs Cas9 H840A nickase to nick the target DNA strand. Molecular mechanism of PE governs by Cas9 H840A nickase with an H840A substitution, inactivating the NHH domain to induce single-stranded nicks via the RuvC domain (Shao et al., 2018). In very next, M-MLV reverse transcriptase transcribes RNA templates into DNA. prime editing guide RNA (pegRNA) provides a primer-binding site (PBS) and a reverse transcriptase template, ensuring precise insertion, deletion, or base alteration without requiring donor templates or DSBs (Oh et al., 2022). In this way, mismatch repair mechanism (MMR) incorporates the new sequence into the genome (Zhu et al., 2020; Yan et al., 2024). Prime editors have garnered significant attention for their potential to repair genetic diseases, develop therapeutic compounds, and redirect plant development and signaling cascades, ultimately improving genome performance. However, prime editing faces limitations due to DNA mismatch repair (MMR), which can lead to insertions or deletions (indels), negatively impacting editing accuracy and efficiency (Ahmad et al., 2023). Despite these challenges, the editing efficiency of prime editors has driven their evolution toward customizable systems that enhance expression of genetic traits under various conditions (Tingting et al., 2023).

Several innovative prime editing systems, (**Table 1**) have been developed to improve functionality and minimize off-target effects. These systems, collectively referred to as prime plant editors (PPE) (**Figure 4**), include variants such as PE1-5, NPE, epegRNA, dual-pegRNA, TWIN-PE, enpPE2, and ePPE (Zeng et al., 2024). The first prime editor (PE1) linked wild-type moloney murine leukemia virus (M-MLV-RT) reverse transcriptase to the Cas9 H840A nickase at the C-terminal domain, achieving notable editing efficiency (Grünewald et al., 2023). PE2 system introduced five amino acid mutations in the M-MLV-RT enzyme, enhancing PE1 functionality and producing the Cas9-H840A-M-MLV-RT complex with improved editing efficiency (Zhao et al., 2023). The challenges of MMR were halted by PE3 which introduce an additional single guide RNA (sgRNA) to direct the Cas9 nickase to nick the original DNA strand near the editing site. Although this approach enhanced repair, it also increased off-target effects (Chen and Liu, 2023). The system PE4 was variant of PE2, that incorporated an additional plasmid encoding a dominant-negative MLH1, which suppressed endogenous MMR by knocking out MLH1, thus enhancing editing efficiency and reducing off-target effects (Truong et al., 2024). PE5 was then introduced relying on PE3 and PE4, that further minimized MMR-related issues and off-target effects while achieving higher editing efficiency (Park et al., 2024). NPE (Nuclease Prime Editor) relies on a Cas9 nuclease requiring only one pegRNA, enabling double-stranded DNA nicking with high stability and efficiency, unlike PE3, which uses a double-nick approach (Rahimi et al., 2024). Engineered pegRNA (epegRNA) method introduces structural modifications at the 3′ ends of pegRNA to reduce degradation and enhance editing precision. Dual-pegRNA: This approach employs NGG-pegRNA and CCN-pegRNA to simultaneously edit forward and reverse DNA strands, improving accuracy (Nelson et al., 2022). Twin Prime Editing (TWIN-PE) utilizes a single editing protein and two pegRNAs to directly replace double-stranded DNA, bypassing MMR mechanisms (Anzalone et al., 2022). Enhanced Plant Prime Editor 2 (enpPE2) uses composite promoters for pegRNA expression and adjustable editing architecture to improve efficiency in plants. Engineered system (ePPE) degrades the ribonuclease H (RNase H) domain and incorporates viral NC proteins, preventing pegRNA degradation and enhancing editing accuracy (Zong et al., 2022). Prime editing systems enable precise genomic modifications, including base substitutions, insertions, deletions, and inversions, with minimal off-target effects. These features make them ideal tools for trait development in horticultural crops and other plants. Zhong et al, demonstrated the stability, accuracy, and high efficiency of the ePPE strategy for prime editing in plants. However, prime editing is not without limitations. Challenges such as window size, target specificity, molecular influences from other proteins, selection pressure, and the sustainability of edited traits remain. Despite these hurdles, the potential of prime editing to develop agronomic crops with enhanced traits and value-added nutrients holds great promise for addressing global food insecurity (Zhao et al., 2023).

## 6 Multi omics, artificial intelligence and synthetic biology for resilient crops

**Figure 5:**
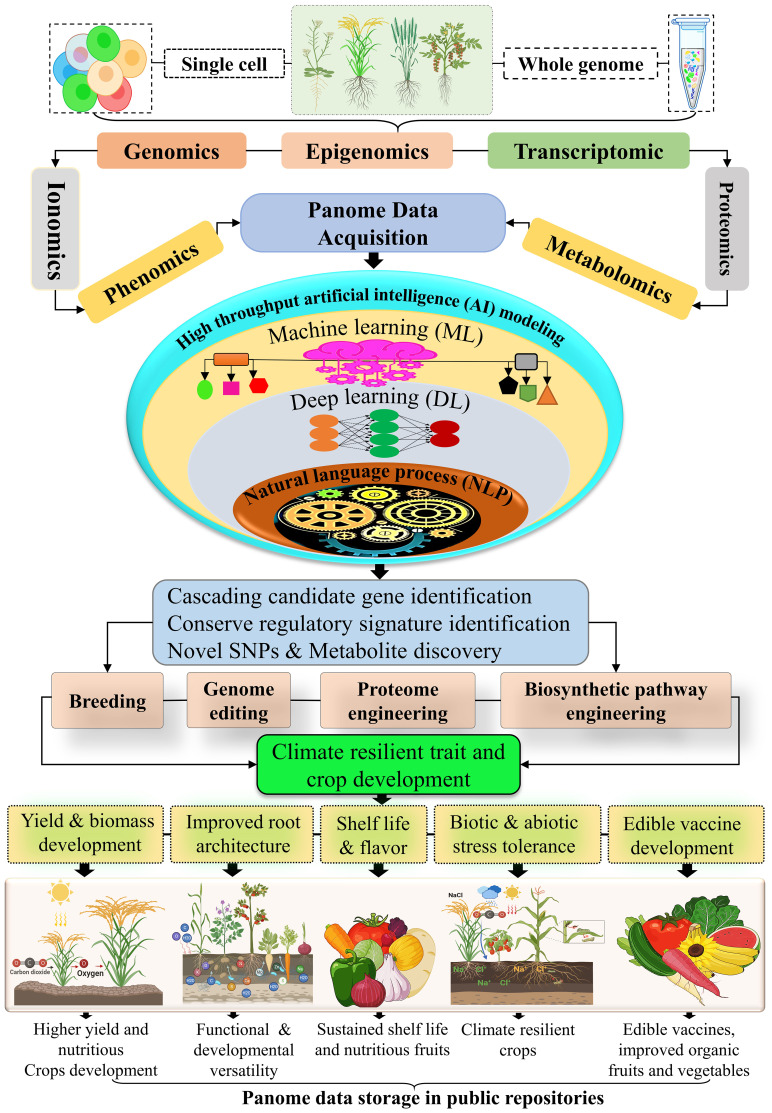
Multiomics integrating genomic, transcriptomic, proteomic, and metabolomic data driven AI and machine learning approaches are transforming crop resilience mechanisms. By analyzing whole-genome and single-cell data, researchers gain comprehensive insights into plant biology. These datasets are processed using advanced omics platforms to identify regulatory elements, cascading genes, SNPs, and metabolites linked to key traits. This information aids in developing climate- and stress-resilient crops with improved yields, taste, texture, shelf life, root efficiency and disease tolerance. High-quality data from these methods are shared in public repositories, supporting global food security and sustainable agriculture.

The advent of synthetic biology has revolutionized genomic research and genetic engineering, offering unprecedented opportunities to manipulate biosynthetic pathways for producing essential compounds and regulating intricate biological processes. The integration of molecular components of networks and pathways, synthetic biology enables the reprogramming of cellular systems to address critical challenges in the development of agronomically relevant and stress-resilient crops (**Figure 5**; Goold et al., 2018). This interdisciplinary field bridges the gap between engineering and biological sciences, facilitating the modular design and rationalization of genetic devices and molecular frameworks. Such innovations aim to develop high-yielding, nutritionally enhanced crops that withstand biotic and abiotic stressors (Lu et al., 2009; Khalil and Collins, 2010). Synthetic biology provides robust tools to decode plant genomes and integrate traits for disease resistance and environmental resilience through multi-gene assembly within plant genomes. This technological advancement has enabled the engineering of complex gene circuits to introduce novel traits in horticultural crops, promoting enhanced productivity and stress tolerance (Yasmeen et al., 2023). Breakthroughs in cost-efficient DNA synthesis, coupled with the development of advanced genetic regulators, such as metabolites, transcription factors, and promoters, have catalyzed the production of crops with superior nutritional profiles, extended shelf life, and improved storage stability under climatic stress (Kaufmann et al., 2010; Li et al., 2024).

The integration of synthetic biology with machine learning and single-cell transcriptomics (scRNA) (**Figure 5**), has further optimized the utilization of metabolic pathways from stress-tolerant organisms like bacteria and algae, enhancing the ability of crops to adapt to environmental challenges and food insecurity. These advancements enable precision design of biological pathways and gene networks, unlocking new avenues for stress resilience, yield improvement, and sustainable agriculture (Long et al., 2022; Sha et al., 2024). High-throughput sequencing technologies, combined with machine learning algorithms, are shaping the future of precision agriculture, fostering global crop resilience (Vazquez-Vilar et al., 2016). Understanding plant stress tolerance mechanisms requires a multi-dimensional exploration at the genome level. High-throughput next-generation sequencing (NGS) approaches, encompassing genomics, epigenomics, transcriptomics, proteomics, metabolomics, and phenomics, have advanced the identification of stress-responsive genomic variants, including substitutions, insertions, deletions, and copy number variations (Kwoji et al., 2023). These multi-omics and AI-driven methodologies elucidate the intricate hierarchies and functional networks required for developing climate-resilient crops (Raza, 2020). Genomics involves deciphering genome-scale regulatory pathways, revealing the influence of intergenic and “dark matter” regions on stress responses (Purugganan and Jackson, 2021). Epigenomics highlights reversible modifications, such as DNA methylation and histone modification, that regulate gene expression without altering the DNA sequence, maintaining critical physiological processes like DNA repair under stress (Luo et al., 2020).

Transcriptomics focuses on RNA-seq analysis to monitor dynamic transcriptome changes under stress, unraveling the cascade of signals that drive defense mechanisms and physiological adaptations. Advanced RNA sequencing technologies facilitate genome-wide profiling of coding and non-coding RNAs, regulatory regions, and enhancers, contributing to a deeper understanding of molecular pathways involved in plant development (Zhou et al., 2022). Proteomics provides insight into cellular states during stress, detailing post-translational modifications, protein-protein interactions, and regulatory networks essential for stress adaptation and developmental processes (Derbyshire et al., 2022). Metabolomics investigates the diversity of primary and secondary metabolites, revealing their roles in stress resilience, including pathways like shikimate, acetate-malonate, and the TCA cycle, which synthesize compounds such as alkaloids, terpenoids, and phenolics. These metabolites, along with exogenous applications of compounds like proline, tryptophan, and glycine betaine, improve crop productivity and stress tolerance (Nowicka et al., 2018; Mohammadzadeh et al., 2022). Phenomics, driven by AI, captures high-throughput phenotypic data to link stress-induced molecular changes with observable traits. Advanced AI models enable precise phenotypic predictions, facilitating the breeding of resilient crops (Harfouche et al., 2023; Zavafer et al., 2023). Integration of scMulti-omics allows simultaneous quantification of genomic, transcriptomic, and proteomic data, enabling the dissection of complex molecular mechanisms underlying environmental resilience (Cao and Gao, 2022).

Lastly, ionomics explores the role of nutrient and ion homeostasis in stress adaptation. High-throughput AI-driven approaches identify biomarkers and cascading pathways involved in ion transport, metal homeostasis, and rhizosphere interactions, paving the way for genetic innovations in nutrient-efficient and stress-tolerant crop development (Xu et al., 2022). These synergistic advancements in synthetic biology, omics technologies, and artificial intelligence have the potential to revolutionize agricultural productivity, ensuring food security while addressing the challenges posed by climate change and environmental stresses. By enhancing the precision and scalability of crop improvement strategies, these technologies hold promise for developing resilient, high-yielding crops capable of thriving in the face of global agricultural challenges.

## 7 Conclusions and future perspective

Biotechnology is revolutionizing agriculture, offering transformative solutions to pressing global challenges like food security and climate resilience. Advances in gene editing, such as CRISPR, enable precise trait modifications, while multi-omics approaches and artificial intelligence (AI) are unlocking key regulatory networks and stress-tolerant traits. These innovations promise to deliver nutrient-rich, environmentally resilient and high-yield crops capable of thriving under extreme environmental conditions. However, significant challenges remain. Public skepticism, regulatory hurdles, and ecological uncertainties still hinder the widespread acceptance of gene-edited crops. Furthermore, the underrepresentation of diverse crop species and the limited exploration of biotechnological interactions with complex environments constrain global impact. Addressing these gaps requires a balanced approach that combines cutting-edge technologies with traditional agricultural practices and localized strategies. The future lies in integrating AI-driven analytics with multi-omics data to identify pivotal molecules and pathways essential for crop resilience. This synergy will accelerate crop development tailored to regional needs while fostering global food security. Equally important is engaging the public to build trust in biotechnology and ensuring innovations are accessible and sustainable. By addressing current limitations and fostering interdisciplinary collaboration, biotechnology has the potential to reshape agriculture, creating resilient, productive, and climate-adapted crops to meet the demands of a growing population. The innovative and environmentally conscious methods ensuring agricultural advancements to benefit both humanity and the planet are critical need of future.
